# Supplementary material for: Microbial genomes from non-human primate gut metagenomes expand the primate-associated bacterial tree of life with over 1000 novel species
Source: Genome Biol. 2019 Dec 28;20:299. doi: 10.1186/s13059-019-1923-9 (PMC6935492; doi:10.1186/s13059-019-1923-9)
Supplement: Supplementary file 2 — Additional file 2: Figure S1. World map reporting NHP metagenomic samples considered in this study together with host and country information. Figure S2. Phylogenetic tree of the Bacteroidetes phylum (uncollapsed version of the tree in Fig. 4b). Figure S3. Comparison between the phylogeny of the host species surveyed in this study and the one of FGB 4487, the only FGB spanning three out of the four host clades. Dashed lines link each MAG of the FGB 4487 tree with the host it was retrieved from, thus showing that genetically close hosts tend to harbor genetically similar bacterial strains. Figure S4. Phylogenetic tree of the Elusimicrobia phylum (uncollapsed version of the tree in Fig. 4c). Figure S5. KO presence/absence profile in Treponema MAGs recovered from both stool and oral cavity samples. Only KOs related to metabolism and present in at least 20% and less than 80% of samples are reported. [file 13059_2019_1923_MOESM2_ESM.pdf]

Supplementary Figures

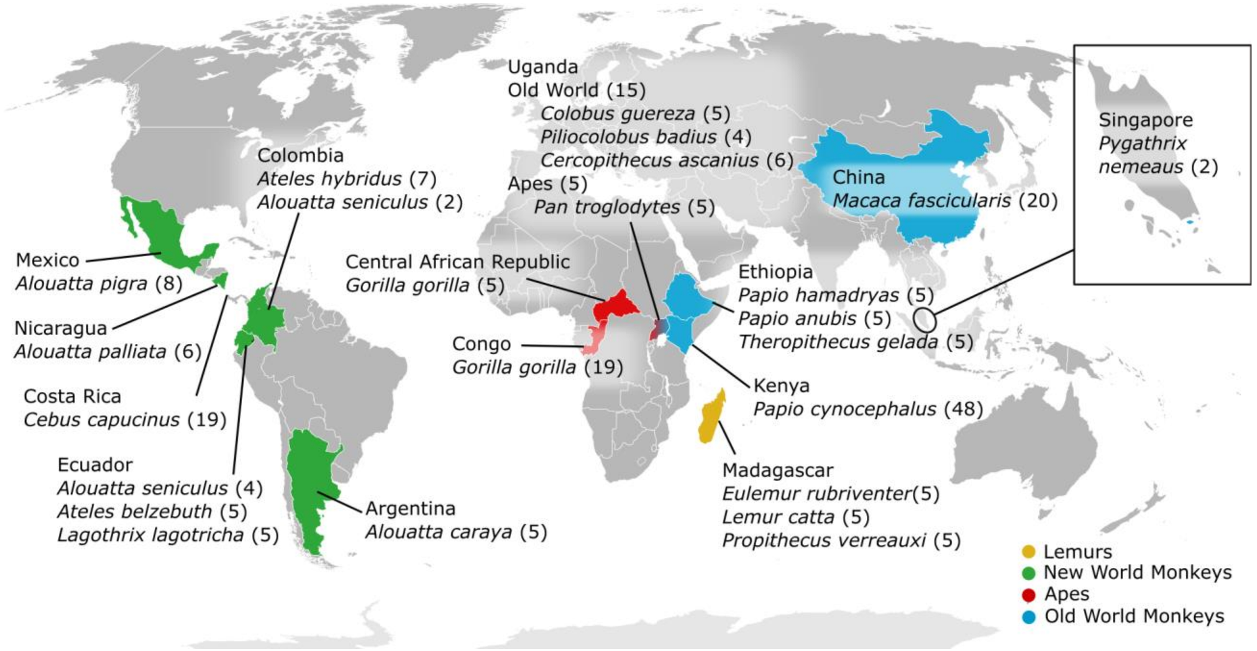

Additional file 2: Figure S1. World map reporting NHP metagenomic samples considered in this study together with host and country information.

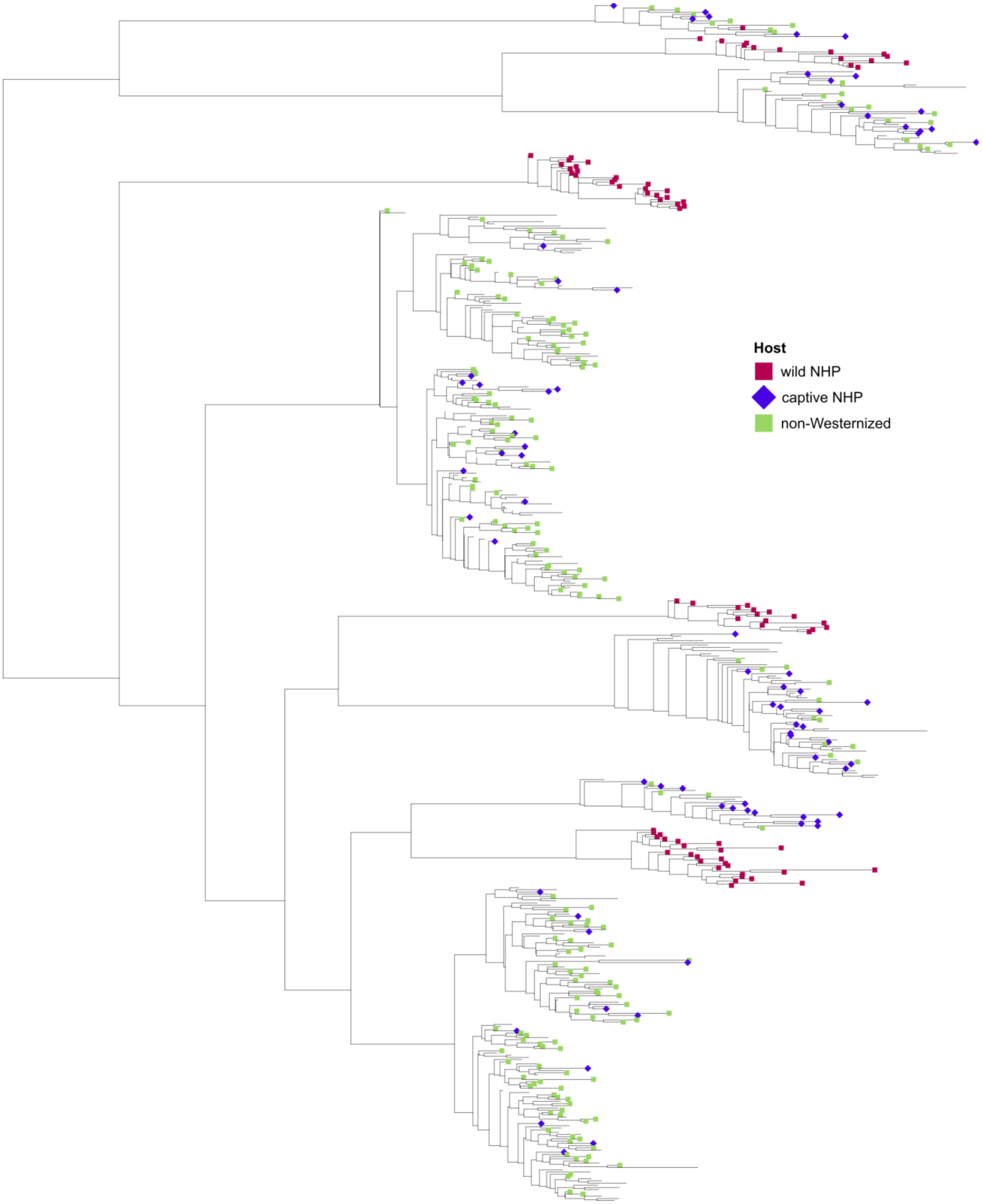

Additional file 2: Figure S2. Phylogenetic tree of the Bacteroidetes phylum (uncollapsed version of the tree in **Figure 4B**).

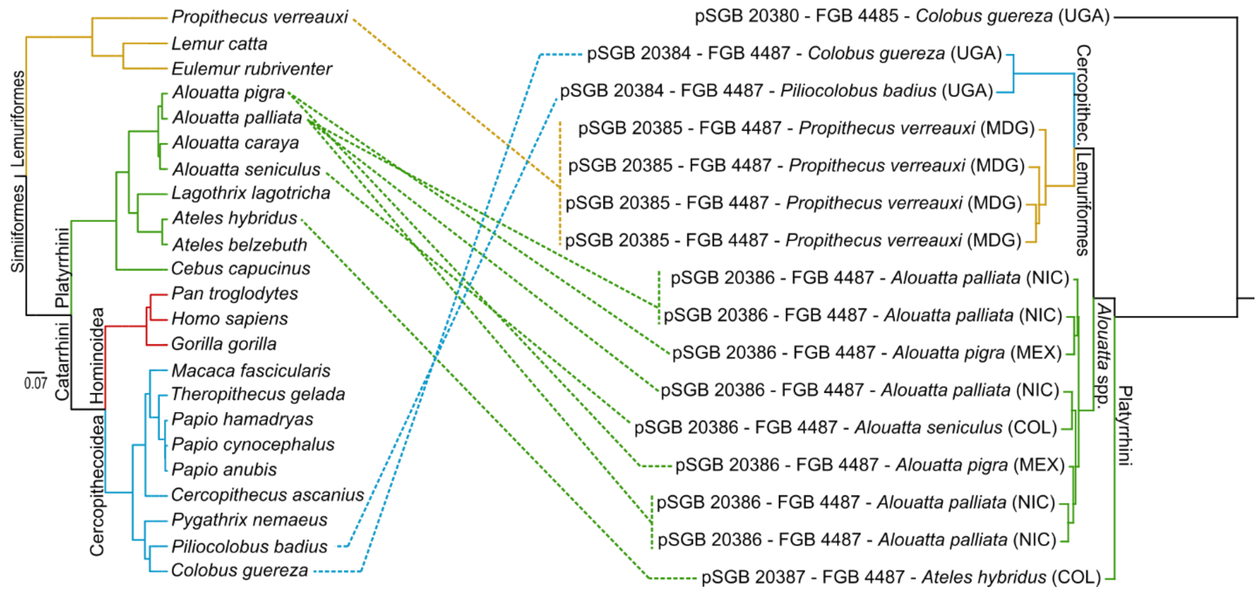

Additional file 2: Figure S3. Comparison between the phylogeny of the host species surveyed in this study and the one of FGB 4487, the only FGB spanning three out of the four host clades. Dashed lines link each MAG of the FGB 4487 tree with the host it was retrieved from, thus showing that genetically close hosts tend to harbor genetically similar bacterial strains.

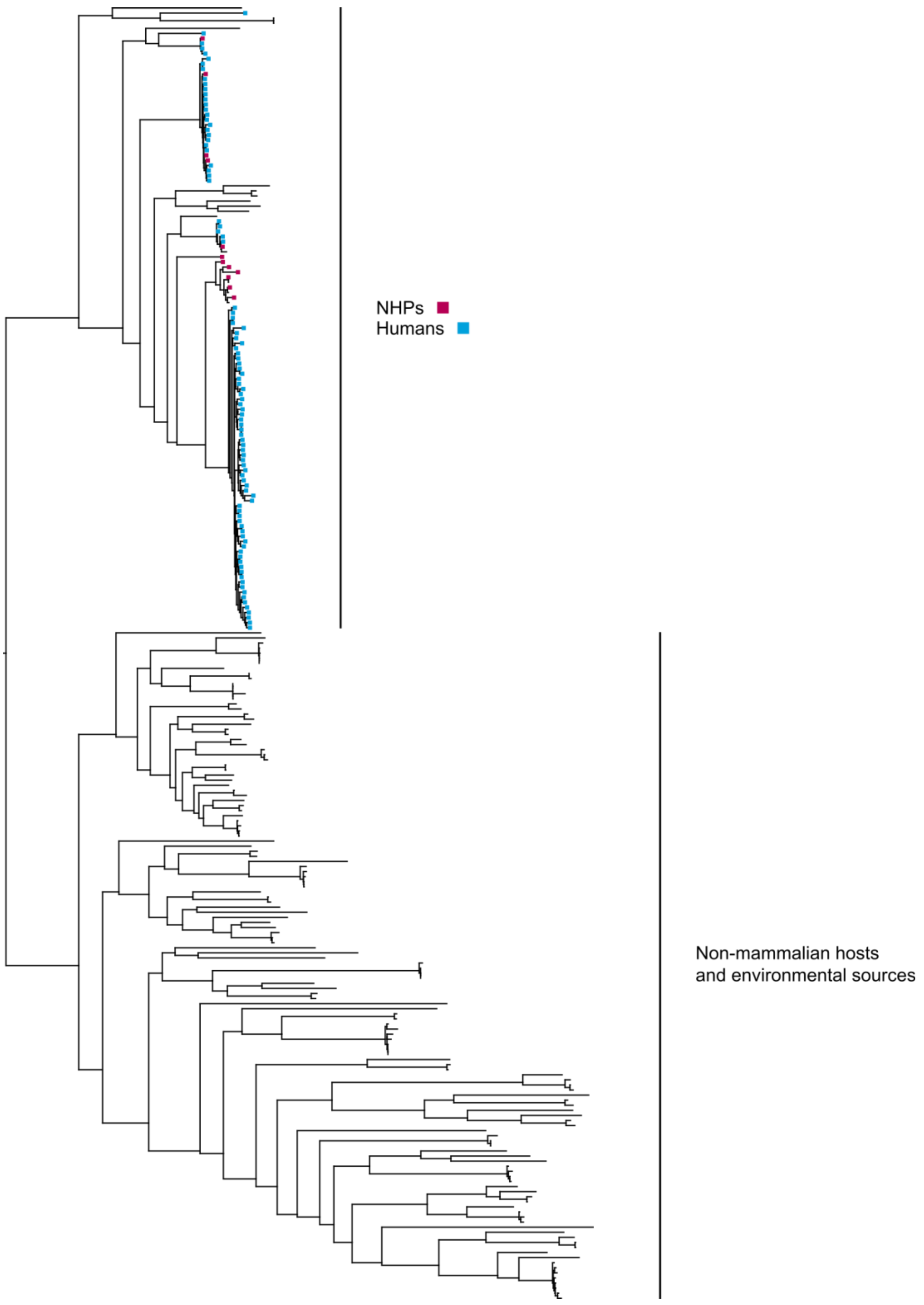

Additional file 2: Figure S4. Phylogenetic tree of the Elusimicrobia phylum (uncollapsed version of the tree in **Figure 4C**).

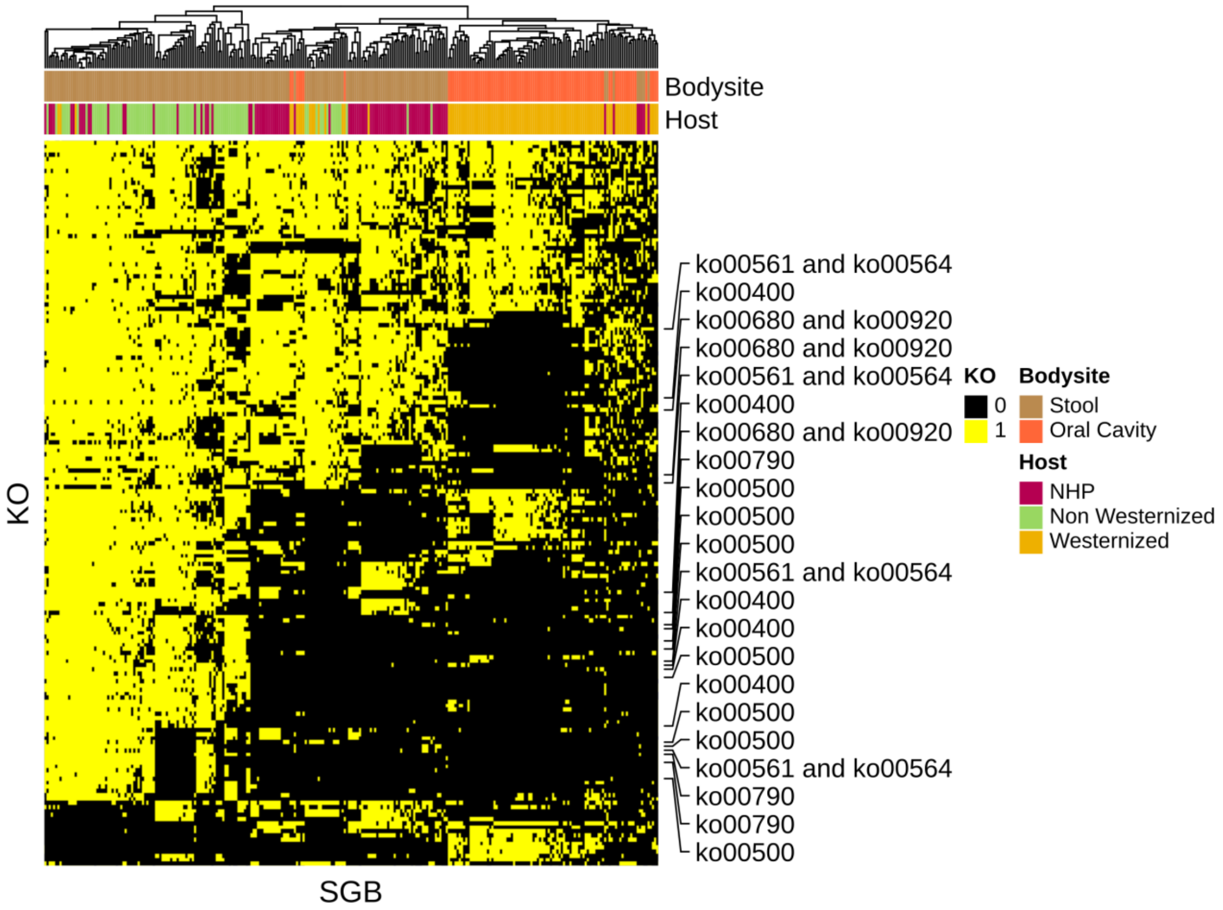

Additional file 2: Figure S5. KO presence/absence profile in *Treponema* MAGs recovered from both stool and oral cavity samples. Only KOs related to metabolism and present in at least 20% and less than 80% of samples are reported.
